# Age de début de la presbytie chez le sujet noir camerounais

**DOI:** 10.11604/pamj.2019.32.162.15808

**Published:** 2019-04-09

**Authors:** Stève Robert Ebana Mvogo, Viola Andin Dohvoma, José Stéphanie Ngassam Fangwa, Caroline Mvilongo Tsimi, Marie Evodie Akono Zoua, Marie Blanche Nguena, Epee Emilienne, Ellong Augustin, Ebana Mvogo Côme

**Affiliations:** 1 Département d'Ophtalmologie-ORL, Faculté de Médecine et des Sciences Biomédicales, Université de Yaoundé I, Yaoundé, Cameroun; 2 Hôpital Gynéco-Obstétrique et Pédiatrique de Douala (HGOPED), Douala, Cameroun; 3 Hôpital Central de Yaoundé (HCY), Yaoundé, Cameroun; 4 Faculté de Médecine et des Sciences Pharmaceutiques de l'Université de Douala, Douala, Cameroun; 5 Hôpital Général de Douala (HGD), Douala, Cameroun

**Keywords:** Age, presbytie, cycloplégie, Cameroun, Age, presbyopia, cycloplegia, Cameroon

## Abstract

**Introduction:**

La prescription d'une addition dans notre pratique n'est pas rare chez les patients de moins de 40 ans. Nous avons recherché le besoin d'une addition après étude objective de la réfraction et partant déterminer l'âge moyen de début de la presbytie.

**Méthodes:**

Nous avons mené une étude transversale et descriptive à l'Hôpital Central de Yaoundé durant trois mois chez les patients âgés de 35 à 45 ans. Ils ont tous bénéficié d'une étude objective de la réfraction sous cycloplégie. La correction de la vision de loin était faite sous cycloplégie, alors que l'addition en vison de près était évaluée deux jours plus tard lorsque la cycloplégie n'était plus effective. La prescription probable d'une addition à la correction de loin devait être réalisée 2 jours après la cycloplégie. L'analyse statistique a été faite avec le logiciel IBM SPSS 20.0.

**Résultats:**

Nous avons examiné 55 patients soit 110 yeux. L'âge moyen des patients était de 41,87±2,5 ans, avec un sex-ratio de 0,28 en faveur des femmes. L'astigmatisme hypermétropique était l'amétropie la plus fréquente avec 58,2% de cas suivi de l'hypermétropie avec 24,6%. Nous n'avons retrouvé aucun sujet myope. L'âge moyen de début de la presbytie dans cette tranche d'âge était de 43,2±1,7 ans. Après correction objective en vision de loin, aucun patient n'avait besoin d'une addition avant 40 ans. Par ailleurs, 4 patients sur 10 avec une amétropie hypermétropique n'ont pas besoin d'addition avant 45 ans. La prescription d'une addition était liée de façon significative à l'âge.

**Conclusion:**

L'âge moyen de début de la presbytie est de 43,2±1,7 ans. Avant 45 ans, toute prescription d'une addition doit être précédée d'une étude objective de la réfraction.

## Abstract (en)

**Introduction:**

Addition in patients of less than 40 years is not uncommon in our practice. We investigated the need for addition after objective refraction and thus determine the average age of onset of the presbyopia.

**Methods:**

We conducted a cross-sectional descriptive study of patients aged 35-45 years at the Central Hospital of Yaoundé over a period of three months. All patients underwent cycloplegic refraction. Distance visual acuity was corrected after instillation of cycloplegic eye drops while near addition was assessed two days later when the cycloplegia was no longer effective. Possible additional distance correction was done 2 days after cycloplegia. Statistical analysis was done using IBM SPSS 20.0 software.

**Results:**

We examined 55 patients, 110 eyes. The average age of patients was 41.87±2.5 years, the sex-ratio was 0.28. There was a female predominance. Hypermetropic astigmatism was the most common ametropia (58.2% of cases) followed by hyperopia (24.6%).No patient was myopic. the average age of presbyopia onset in this age group was 43.2±1.7 years. After objective correction of distance visual acuity, no patient needed for addition before the age of 40. Moreover, 4 out of 10 patients with hypermetropic ametropia did not need addition before the age of 40. Addition was significantly associated with age.

**Conclusion:**

The average age of presbyopia onset is 43.2±1.7 years. Before the age of 45, addition must be preceded by objective refraction.

## Introduction

La presbytie est une amétropie dynamique qui résulte de la perte progressive du pouvoir accommodatif de l'œil atteint [1]. La presbytie est une cause importante de déficience visuelle évitable à travers le monde. Brien *et al*estimaient à 1,04 milliards le nombre de presbytes à travers le monde en 2005. Selon ces auteurs, 94% des presbytes non corrigés vivent dans les pays en voie de développement [2]. La presbytie va induire un handicap visuel important en vision de près chez le sujet emmétrope ou emmétropisé. Il est alors indispensable d'adjoindre une addition à la vision de loin du sujet presbyte. Les verres correcteurs prescrits, qu'ils soient progressifs ou bifocaux restent difficilement accessibles pour la grande majorité des patients dans notre milieu. Du reste, il n'est pas rare de voir des patients avec des verres progressifs prescrits avant l'âge de 40 ans alors même que l'emmétropisation effective n'est pas obtenue. Murphy et al situe l'âge moyen de début de la presbytie à 40ans chez le sujet emmétrope [3]. Jain et al retrouvaient un âge moyen de début de la presbytie de 39,2 ans; 40,1 ans et 40,8 ans respectivement chez les hypermétropes, emmétropes et myopes [4]. Afin de pouvoir améliorer la prise en charge de la presbytie dans notre milieu, nous avons voulu déterminer de façon objective l'âge de début de la presbytie chez le sujet noir camerounais.

## Méthodes

Nous avons mené une étude transversale et descriptive à l'Hôpital Central de Yaoundé du 1^er^ février au 30 avril 2017. Nous avons inclus tous les nouveaux patients âgés de 35 à 45 ans mélanodermes et camerounais ne lisant pas P2 venus en consultation. Tous les patients chez qui l'examen clinique relevait une cause organique pouvant expliquer la baisse de l'acuité visuelle de près ont été exclus. Les données socio démographiques des patients, les antécédents, ainsi que les signes fonctionnels ont été recherchés pendant l'interrogatoire. Tous ont bénéficié d'un examen ophtalmologique complet comprenant un examen oculomoteur. Cet examen était complété par un examen des annexes de l'œil, du segment antérieur et du fond d'œil. La pression intraoculaire au tonomètre à air pulsé a été systématiquement mesurée. L'acuité visuelle de loin (AVL) a été mesurée avec l'échelle de Monoyer en ambiance mésopique. Le sujet était en position assise et à une distance de 5 mètres de l'échelle. La mesure de l'acuité visuelle de près (AVP) a été mesurée avec l'échelle de Parinaud, le sujet en position assise tenait l'échelle à 33 cm de lui. La mesure de l'acuité visuelle de loin et de près était monoculaire et l'œil droit était toujours testé en premier. Tous ont bénéficié d'une étude objective de la réfraction. La cycloplégie était obtenue après instillation d'une goutte de Tropicamide 0,5% alternativement avec une goutte de chlorhydrate de Cyclopentolate 0,5% toutes les 5 minutes pendant 25 minutes. La réfraction a été réalisée par le refractomètre automatique (KR-8100 de marque Topcon). La prescription d'une addition pour la vision de près était requise pour ceux des patients qui ne parvenaient pas à lire P2, 48heures après la cycloplégie avec la correction optique optimale de loin. Nous avons analysé: 1) l'âge, 2) le sexe, 3) le niveau d'étude, 4) le type d'amétropie, 5) l'acuité visuelle de près avant et après une correction de loin, et si nécessaire après une addition. Nous avons classé les amétropies après cycloplégie en hypermétropie, myopie, astigmatisme hypermétropique, astigmatisme myopique et astigmatisme mixte. Nous avons considéré comme presbyte tout patient ne lisant pas P2 à l'échelle de Parinaud après une emmétropisation en vision de loin. L'analyse des données a été effectuée à l'aide du logiciel IBM SPSS version 20.0. Les tableaux et graphiques ont été faits à l'aide du logiciel Microsoft Excel 2016. Nous avons utilisé les tests de Chi 2 et Fisher pour comparer les proportions. La comparaison des moyennes dans les groupes a été faite à partir du test ANOVA. Le seuil de significativité était p< 0,05.

## Résultats

Nous avons colligé au total cinquante-cinq patients et examiné 110 yeux. On notait 43 patients de sexe féminin (78,2%) et 12 patients de sexe masculin (22,8%). Le sex-ratio est de 0,28 en faveur des femmes. L'âge moyen des patients était de 41,87±2,5 ans. Les patients ont été répartis selon leur âge en deux tranches (Tableau 1). La tranche d'âge allant de 40 à 45 ans était la plus représentée avec 47 patients soit 85,5%. Les patients ayant un niveau d'étude secondaire étaient les plus nombreux (49,1%), puis suivaient les patients avec un niveau d'étude supérieur soit 34,5% des malades (Tableau 1). Après étude de la réfraction, les amétropies retrouvées pour les 110 yeux examinés sont présentées dans le Tableau 2. L'astigmatisme hypermétropique représentait 58,2% des amétropies soit 64 yeux, suivi par l'hypermétropie avec 24,6% soit 27 yeux. Aucun cas de myopie n'a été retrouvé. Nous n'avons pas retrouvé de différence dans la répartition des amétropies selon l'âge (p=0,2), le sexe (p=0,41) ou la latéralité (p=0,93). L'âge moyen des patients ne lisant pas P2 avant réfraction était de 41,87±2,5 ans. Après réfraction sous cycloplégie, l'âge moyen des patients incapables de lire P2 était de 43,2±1,2 ans. Aucun patient n'avait besoin d'une addition avant 40ans après réfraction sous cycloplégie. Entre 40 et 45 ans, 61,7% des patients (soit 29 patients sur 35) étaient presbytes. L'âge moyen de début de la presbytie chez les hommes était de 43,38±1,4 ans contre 43,10±1,8 ans chez les femmes (p=0,9). L'âge moyen de début de la presbytie chez les sujets ayant un niveau d'étude primaire, secondaire et supérieur est de 43,80±2,2 ans, 42,92±1,7 ans et 43,18±1,5 ans respectivement. Il n'y avait pas de différence statistiquement significative entre l'âge de début de la presbytie et le niveau d'étude (P=0,62). L'âge moyen de début de la presbytie chez les sujets hypermétropes et astigmates hypermétropes est de 43,72±1,7 ans et 42,88±1,7 ans. Cet âge chez les sujets astigmates myopes et mixtes était de 43,33±0,6 ans et 43±1,2 ans respectivement. La différence n'était pas statistiquement significative entre les différentes amétropies statiques retrouvées (P=0,09).

**Tableau 1:** Répartition socio-démographique des patients (N=55)

Paramètres	Effectif	Pourcentage (%)
**Age**		
35-39ans	8	14,5
40-45ans	47	85,5
**Niveau d’étude**		
Primaire	9	16,4
secondaire	27	49,1
supérieur	19	34,5

**Tableau 2:** Types d’amétropie

Amétropies	Effectif	Pourcentage (%)
Hypermétropie	27	24,6
Myopie	0	0
Astigmatisme hypermétropique	64	58,2
Astigmatisme myopique	15	13,6
Astigmatisme mixte	4	3,6
Total	110	100

## Discussion

Dans notre série, l'âge moyen des patients colligés est de 41,87±2,5 ans avec un sex-ratio de 0,28 en faveur des femmes. Naidoo *et al.* en Afrique du Sud trouvent un âge moyen de 52 ans [5]. Umar *et al.* dans une série au Nigeria trouvent un âge moyen de 53,59 ans [6]. Dans une précédente série camerounaise, Bella *et al.*trouvent un âge moyen de 44,5 ans avec une prédominance du genre féminin. Il faut souligner que l'âge moyen varie selon les critères de sélection et le type de population. Dans notre série, seuls les patients d'au moins 35 ans et d'au plus 45 ans étaient retenus, ce qui pourrait expliquer que l'âge moyen des patients de notre série soit plus faible que celui des autres séries africaines [5-7]. Après étude de la réfraction sous cycloplégie, nous retrouvons comme présenté dans le Tableau 2, 58,2% d'astigmatismes hypermétropiques suivis des hypermétropies avec 24,6% des yeux examinés. Ces résultats sont semblables au profil des amétropies statiques de la population camerounaise où Ebana *et al* trouvent une prédominance de l'hypermétropie et de l'astigmatisme hypermétropique [8]. Par contre, nous n'avons pas trouvé de sujets myopes. Les symptômes liés à la presbytie apparaissent bien plus tardivement chez les myopes comparés aux hypermétropes. Katz l'expliquerait par le fait que la plupart des sujets myopes peuvent travailler de près sans correction; de plus ils sont souvent sous corrigés de loin et gardent plus longtemps la capacité à lire de près [9]. Abraham *et al.*trouvent que l'amplitude d'accommodation entre 35 et 44 ans est plus grande chez les myopes que les hypermétropes ou les emmétropes. Même si les patients myopes perdraient progressivement cet « avantage » dès l'âge de 45 ans [10]. La presbytie n'apparaitrait donc chez le myope qu'après 45 ans.

L'âge moyen de début de la presbytie est de 43,2 ans dans notre série. Après réfraction sous cycloplégie, nous ne retrouvons aucun patient presbyte avant 40 ans. Une addition a été par contre prescrite chez 61,7% des patients de plus de 40 ans. Avant 45 ans, 4 patients sur 10 n'ont pas besoin d'addition. Nous ne trouvons pas de myope dans notre série. Cette différence rapportée à l'âge est statistiquement significative (p=0,007). Pour Yazdani *et al.*, l'utilisation de cycloplégiques permet de réaliser une étude objective de la réfraction et d'éviter les sous et/ou sur corrections lors de la correction pour la vision de loin [11]. On peut ainsi minimiser plus facilement les effets d'une accommodation résiduelle [11,12]. Il faudrait donc systématiquement prescrire une cycloplégie avant 45 ans afin d'obtenir une emmétropisation, la plus objective possible et partant de pouvoir éviter la prescription d'une addition erronée au malade. Dans notre série, le genre et le niveau d'étude n'avait pas d'influence sur l'âge moyen de début de la presbytie. C'est aussi le constat que font Lu *et al.* en Chine [13]. Par contre, Burke *et al.* en Tanzanie trouvent une prévalence élevée de la presbytie chez les femmes, chez les patients avec un niveau d'éducation élevé, et chez ceux résidents en milieu urbain [14]. Il est admis que la presbytie est précoce chez les hypermétropes [15]. Même si, la presbytie commençait plutôt chez les patients astigmates hypermétropes dans notre travail (42,88±1,7 ans), nous n'avons pas retrouvé de différence significative entre les différentes amétropies retrouvées. Ce constat serait dû à la recherche d'un défaut en vision de près après emmétropisation préalable de loin chez tous les malades. En d'autres termes, cette emmétropisation systématique réduirait fortement l'influence du type d'amétropie sur l'âge de survenue de la presbytie. Toutefois, la presbytie semble être plus précoce dans les amétropies astigmates.

**Limites**: ce travail présente quelques limites. Il s'agit d'une étude transversale et monocentrique intra hospitalière. La taille de l'échantillon est faible. Les résultats retrouvés ne peuvent pas s'appliquer à toute la population camerounaise. Il serait souhaitable de mener une étude à plus grande échelle.

## Conclusion

L'âge moyen de début de la presbytie est de 43,2±1,7 ans. Nous n'avons pas de différence retrouvé une influence du genre, du niveau d'étude ou des amétropies retrouvées sur l'âge de début de la presbytie. Par contre après une étude objective de la réfraction, nous ne notions aucune presbytie avant 40 ans. Avant 45 ans, toute prescription d'une addition doit être précédée d'une étude objective de la réfraction.

### Etat des connaissances actuelles sur le sujet

- La presbytie est une amétropie dynamique qui apparait après 40 ans;
- Elle est précoce chez les sujets hypermétropes.

### Contribution de notre étude à la connaissance

- Avant 45 ans, toute prescription d'une addition doit être précédée d'une étude objective de la réfraction;
- L'âge moyen de début de la presbytie est de 43 ans chez le mélanoderme camerounais;
- La presbytie apparaît plus précocement dans les amétropies astigmates comparées aux amétropies stigmates.

## Conflits d'intérêts

Les auteurs ne déclarent aucun conflit d'intérêts.
